# Comparison of Efficacy and Safety of Tenofovir and Entecavir in Chronic Hepatitis B Virus Infection: A Systematic Review and Meta-Analysis

**DOI:** 10.1371/journal.pone.0098865

**Published:** 2014-06-06

**Authors:** Weixia Ke, Li Liu, Chi Zhang, Xiaohua Ye, Yanhui Gao, Shudong Zhou, Yi Yang

**Affiliations:** Department of Epidemiology and Biostatistics and Guangdong Key Lab of Molecular Epidemiology, School of Public Health, Guangdong Pharmaceutical University, Guangzhou, Guangdong, China; MOE Key Laboratory of Environment and Health, School of Public Health, Tongji Medical College, Huazhong University of Science and Technology, China

## Abstract

**Objective:**

Tenofovir (TDF) and entecavir (ETV) are both potent antiviral agents for the treatment of chronic hepatitis B virus (HBV) infection. Multiple studies have compared efficacy and safety of these two agents, but yielded inconsistent results. Hence, we conducted a meta-analysis to discern comparative efficacy and safety.

**Methods:**

Published data relevant to a comparison of TDF and ETV used in HBV were included. HBV DNA suppression rate, ALT normalization rate, and HBeAg seroconversion rate at 24 weeks and 48 weeks were reviewed. Drug safety profiles and resistance were also discussed.

**Results:**

Seven articles met entry criteria. Four and six articles included data for 24 and 48-week HBV DNA suppression rates, respectively, and no significant differences for the rates between the two drugs were found in chronic HBV patients (TDF vs. ETV: relative risk [RR] = 1.10, 95% CI = 0.91–1.33 and RR = 1.07, 95% CI = 0.99–1.17 for 24 weeks and 48 weeks, respectively). For the ALT normalization rate (three studies for 24 weeks, four articles for 48 weeks) and HBeAg seroconversion rate (two and four studies for 24 weeks and 48 weeks, respectively), no difference was observed between TDF and ETV. Additionally, no significant distinction in short term safety was found for CHB patients.

**Conclusions:**

TDF and ETV are similarly effective and safe in chronic HBV patients after 24 weeks and 48 weeks of anti-viral therapy. Nevertheless, the long-term efficacy and safety of TDF and ETV should be monitored in prolonged therapy.

## Introduction

Chronic hepatitis B virus (HBV) infection remains a serious global health concern. Currently, approximately 2 billion people have been infected with HBV, and over 350 million are suffering from chronic hepatitis B (CHB) worldwide [1]. Cirrhosis, live failure, and/or hepatocellular carcinoma (HCC) are expected to develop in 15%–40% of patients with CHB without appropriate treatment [2], and approximately 1 million patients die annually of cirrhosis, liver failure, and HCC as a result of chronic HBV infection [3]. Therefore, the main goal of treatment of chronic infection is to effectively suppress viral replication, preventing liver disease, progression to cirrhosis, liver failure, and HCC [4]–[6]. Effective antiviral therapy via sustained HBV DNA suppression has become a priority research focus for chronic infection [4]–[6]. Available antiviral drugs include immunomodulatory drugs (interferon-alpha and pegylated interferon-alpha) and nucleotide analogue (NAs) polymerase inhibitors (lamivudine [LAM], adefovir [ADV], entecavir [ETV], telbivudine [LdT], and tenofovir [TDF]). Since interferons are expensive, require parental administration, and cause side effects, oral nucleos(t)ide analogues are preferred [7]. Although LAM, ADV, and LdT are approved for the treatment of chronic HBV infection, high rate of resistance has plagued therapeutic use. At present, the two first line nucleoside/ nucleotides are ETV and TDF. ETV is a potent antiviral that effectively suppresses HBV DNA replication. It has a high genetic barrier for resistance in HBeAg-positive and HBeAg-negative patients [8]–[9] with a cumulative resistance probability of 1.2% after 5 years of treatment [10]. However, in lamivudine-refractory patients, the cumulative probability of genotypic ETV resistance developing over 5 years is 51% [11]. TDF is newer and considered a higher efficiency antiviral drug with a high genetic barrier. To date, no evidence exists to show development of resistance to TDF up to 144 weeks of therapy [12]. Moreover, TDF has been demonstrated to be effective in patients with both adefovir and lamivudine failure [13]. TDF is more effective than ETV to achieve rapid viral suppression in HBeAg-positive chronic HBV patients [14]. Additionally, the Bayesian meta-analysis by Woo *et al.* highlighted TDF as the more effective agent for HBeAg-negative patients during the first year of therapeutic intervention [15]. TDF is proposed to be superior to ETV for treating chronic HBV; however, a more promising result was shown by multiple studies claiming that both are similar in both efficacy and safety [16]–[21]. Due to the small sample sizes of past studies and subsequent limited data for comparing the two drugs, a more definitive conclusion is lacking. Herein, we conducted this meta-analysis by integrating published drug-based data to compare efficacy and safety of TDF and ETV and ultimately provide evidence for clinical decisions.

## Materials and Methods

### Literature search

Pubmed/Medline, Web of Science, EMBASE, The Wiley Online library, CNKI, WANFANG database, the Cochrane Central Register of Controlled Trials, and the Cochrane Database of Systematic Review databases were searched for relevant articles through June 30,2013 without language limitation. The search strategy was based on a combination of the key words “chronic hepatitis B virus or HBV or CHB”, “entecavir or ETV”, “tenofovir or TDF”. Reference lists from retrieved documents were also scanned. Two reviewers independently screened citations and abstracts of each article (Wei-xia Ke and Chi Zhang).

### Inclusion and exclusion criteria

The following inclusion criteria were used for this meta-analysis: (1) randomized and non-randomized control trials (included cohort or case-control studies), (2) study population consisting of patients with chronic HBV infection, and (3) intervention therapies of entecavir versus tenofovir monotherapy. The following types of studies were excluded: (1) studies of patients who were co-infected with HIV, HCV, or HDV, (2) studies of patients who adopted combination therapy or sequential therapy, (3) studies of individuals who used of immunomodulatory drugs or other nucleotide analogues within the preceding 6 months, (4) studies not reporting any efficacy measures or not conveying sufficient statistical information, and (5) studies not including either tenofovir or entecavir.

### Efficacy measures

Efficacy was considered for patients 24 and 48 weeks post therapy by considering the following: HBV-DNA level (<400 copies/ml), ALT normalization rate (<40 IU/ml), HBeAg seroconversion rate (HBeAg loss and the appearance of HBe antibody), and drug safety (adverse events, laboratory abnormalities, deaths, tolerability, etc).

### Data extraction

Two authors extracted data independently and recorded the following for each publication: the first author’s name, published year, country of study, time of study (start date and end date), number of patients, details of study design, patient characteristics (average age, gender, etc), treatment doses and duration, and outcome measures performed (described earlier). Where eligible, the authors of articles with insufficient data were contacted. If they did not provide data after contact, those articles were excluded from our meta-analysis.

### Study quality

The two reviewers also assessed methodological quality based on following criteria: (1) Randomized controlled trials (RCTs) were assessed using the QUOROM guidelines and the Jadad scale [22]; (2) non-RCTs must have met the case matched by the patient’s baseline data; (3) selected studies had defined inclusion and exclusion criteria for the study population and a clear definition of treatment responses. Reviewers resolved discrepancies through discussion.

### Statistical analysis

Pooled rates for DNA suppression, ALT normalization, and HBeAg seroconversion were estimated using the inverse variance method. Relative risks (RRs) and 95% confidence intervals (CIs) as metrics of effect size were re-calculated for TDF versus ETV (as reference) in DNA suppression, ALT normalization, and HBeAg seroconversion rates. Inter-study heterogeneity was evaluated by the *χ* ^2^- based Cochran’s Q statistic test and *I^2^* metric, with significance set at *P*<0.10 or *I^2^*>50%. In the absence of significant heterogeneity, the fixed-effect model,using Mantel-Haenszel method was applied to combine results [23]; In other cases, the random-effect method, using DerSimonian and Laird methods was applied [24]. Sensitivity analysis assessed whether a single study significantly affected overall estimates by sequentially removing studies. The Begg’s rank correlation test and Egger’s linear regression test were conducted for assessing publication biases. All statistical analyses were carried out in STATA V11.0, and all *P* values are two-tailed with a significant level at 0.05. This meta-analysis was conducted according to the Preferred Reporting Items for Systematic Reviews and Meta-analyses (PRISMA) statement (Checklist S1) [25].

## Results

### Search results and study characteristics

**Figure 1:**
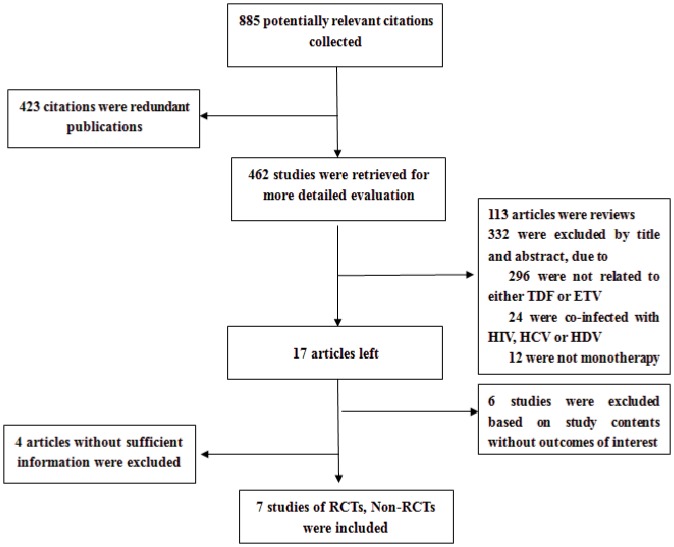
Flow diagram of selecting included studies.

We identified 885 citations *via* electronic searches (Fig. 1). Seven were selected describing treatment of chronic HBV infection involving 844 patients (378 treated with TDF monotherapy and 466 treated with ETV monotherapy). Of these studies, two were RCTs [16]–[17], four were cohort studies [18]–[21], and one was a case-cohort study [14]. The detailed information of included studies is summarized in Table 1.

**Table 1:** Main characteristics of included studies.

Study	Location	Ethnicity	Study design	Sample size		Gender		Age(yrs)	Baseline HBV DNA level	HBeAg (+)/(−)		Status
				TDF	ETV	Male	Female	TDF vs. ETV		TDF	ETV	
Liaw [16]	Worldwide	Asian/White/Other	RCTs	45	22	54	13	Median (range): 52(48–57) vs. 54(47–58)	≥10^3^ copies/ml	14/31	7/15	Non-Naïve, Cirrhosis
Sriprayoon [17]	NA	NA	RCTs	100	100	NA	NA	Mean(SD): 42.4(11.5) vs. 40.8(11.0)	>2,000 IU/ml	52/48	54/46	Naïve, Cirrhosis
Koklu [18]	Turkey	NA	Cohort	72	77	114	35	Mean(SD): 54.2(10.5) vs. 52.4(11.2)	5.4±1.9log copies/ml	9/62	17/60	Non-Naïve, Cirrhosis
Dogan [19]	Turkey	NA	Cohort	65	29	58	36	NA	Detectable	29/36	10/19	Naïve, Cirrhosis
Kurdas [20]	Caucasus	Caucasian	Cohort	20	24	31	13	Mean (SD): 37.75(10.10) vs. 43.63(8.97)	≥6 log copies/ml	6/14	5/19	Naïve, Cirrhosis
Jayakumar [21]	India	NA	Cohort	19	20	35	4	Mean (SD): 34(9.60) vs. 42.15(17.11)	≥10^4^ copies/ml	10/9	15/5	Naïve, CHB
Gao [14]	U.S.	NA	Case Cohort	57	194	96	155	Mean: 41.6 vs. 43.2	>1,000,000 IU/ml	37/20	120/74	Naïve, CHB

Note: NA, not available; TDF, tenofovir; ETV, entecavir; SD, Standard Deviation; RCTs, randomized controlled trials; CHB, chronic hepatitis B.

### Study quality

Two manuscripts [16]–[17] were RCTs. One received Jadad scores of 5 and the other 3 (Table 2). For non-RCTs, all were well-matched based on baseline characteristics and clear definition of treatment response. With exceptions of Gao *et al.* [14] and Kurdas *et al.* [20] non-RCTs had defined inclusion and exclusion criteria for patients (Table 3).

**Table 2:** Methodical assessment of RCT studies.

Study	Adequate sequence genetation	Allocation concealment	Blinding	Incomplete outcome data addressed	Free of selective reporting	Free of other bias	Jadad
Liaw [16]	Yes	Yes	Yes	Yes	Yes	Yes	5
Sriprayoon [17]	Unclear	Unclear	Unclear	Unclear	Unclear	Unclear	3

**Table 3:** Methodical assessment of non-RCT studies.

Study	Case Matched	Well-defined inclusion and exclusion criteria	Clear definition of treatment responses
Dogan [19]	Yes	Yes	Yes
Jayakumar [21]	Yes	Yes	Yes
Koklu [18]	Yes	Yes	Yes
Kurdas [20]	Yes	Only inclusion criteria	Yes

### HBV DNA suppression rate

**Figure 2:**
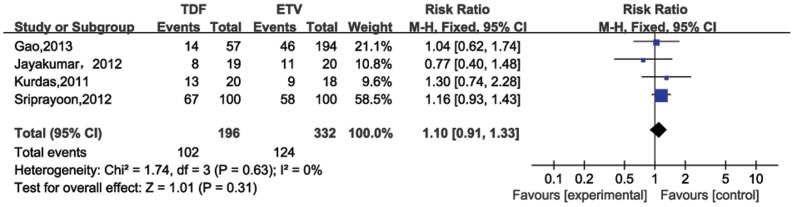
Forest plot for HBV DNA suppression rates 24 weeks post therapy.

**Figure 3:**
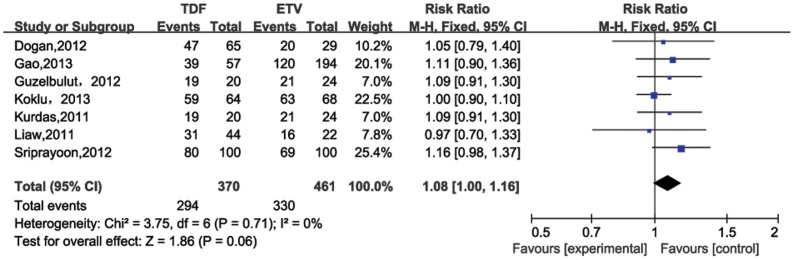
Forest plot for HBV DNA suppression rates 48 weeks post therapy.

For four studies evaluating patients post 24 weeks of therapy [14], [17], [20], [21], the pooled HBV DNA suppression rates were 50% (95%CI = 25%–74%) and 46% (95%CI = 24%–68%) for TDF and ETV, respectively. No significant heterogeneity existed across studies (*P* for heterogeneity = 0.63; I^2^ = 0%). In the fixed-effect model, no significantly difference was determined between TDF and ETV treatment groups in the HBV DNA suppression rate through 24 weeks of treatment (RR = 1.10, 95% CI = 0.91–1.33; Fig.2). Six studies compared HBV DNA suppression rates after 48 weeks of therapy (Fig.3), which were also similar for TDF (80%, 95%CI = 71%–90%) and ETV (76%, 95%CI 63%–88%). No significant heterogeneity was observed (*P* = 0.59, I^2^ = 0%), and no significant difference was also observed in that rate after 48 weeks treatment between the two treatment groups (RR = 1.07, 95%CI = 0.99–1.17). The statistic power for 24 and 48 weeks HBV DNA suppression rates were 92.08% and 85.03%, respectively.

### ALT normalization rate

**Figure 4:**
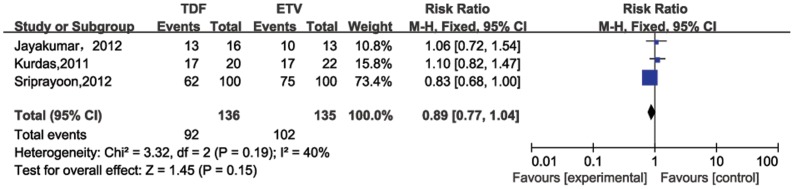
Forest plot for ALT normalization rates 24 weeks post therapy.

**Figure 5:**
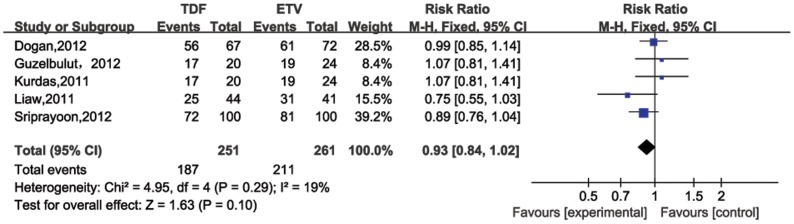
Forest plot for ALT normalization rates 48 weeks post therapy.

Pooled ALT normalization rates from three studies were 75% (95%CI = 59%–91%) and 76%(95%CI = 68%–83%) that included TDF and ETV groups 24 weeks post treatment, respectively. No significant heterogeneity was detected (*P* for heterogeneity = 0.19; *I^2^* = 40%). No significant difference between TDF and ETV for ALT normalization rate was calculated for 24 weeks post treatment (RR = 0.89, 95% CI = 0.77–1.04) (Fig.4). Four studies involved comparing ALT normalization rates after 48 weeks of therapy (Fig.5). The pooled rate for the TDF group (74%, 95%CI = 62%–86%) was similar to that of the ETV group (81.0%, 95%CI = 76%–86%). No significant difference was calculated after 48 weeks treatment between the two drugs (RR = 0.91, 95%CI = 0.83–1.01; *P* for heterogeneity = 0.26, *I^2^* = 26%).

### HBeAg seroconversion rate

**Figure 6:**
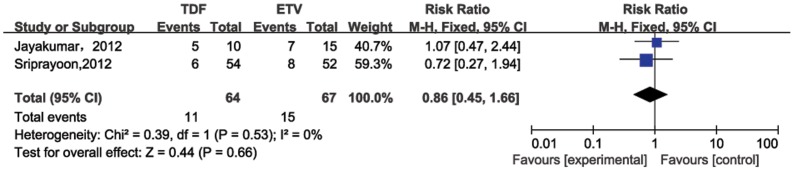
Forest plot for HBeAg seroconversion rates 24 weeks post therapy.

**Figure 7:**
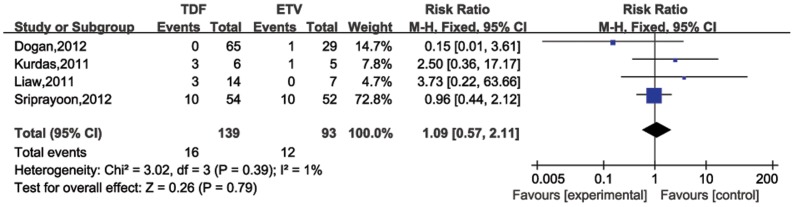
Forest plot for HBeAg seroconversion rates 48 weeks post therapy.

Two studies involved HBeAg seroconversion rates 24 weeks post treatment (Fig.6). Heterogeneity was found to be a concern (*P* for heterogeneity = 0.53; I^2^ = 0%). The results of the two studies indicated that the pooled HBeAg seroconversion rate for the TDF group was 28% (95%CI = −10%–65%) and the ETV group response rate was 29% (95%CI = −12%–59%). The pooled relative risk was 0.86 (95%CI = 0.45–1.66), suggesting no significant difference. Four studies were included to compare HBeAg seroconversion rates at 48 weeks post treatment (Fig.7). The pooled rates of 48 weeks post therapy were similar between TDF (16%, 95%CI = 0%–32%) and ETV (10%, 95%CI = 0%–20%). Heterogeneity was not found (*P* for heterogeneity = 0.39; I^2^ = 1%), and a difference between the two groups was not significant (RR = 1.09, 95%CI = 0.57–2.11).

### Tolerability and Safety of tenofovir and entecavir

Liaw *et al.* [16] found no significant differences between the TDF and ETV treatment groups for both co-primary safety endpoints (tolerability failures and confirmed changes in renal parameters) in decompensated CHB patients. Most adverse events (AEs) and laboratory abnormalities were consistent with decompensated cirrhosis, with few AEs related to these two agents. Sriprayoon *et al.* [17] reported no serious adverse events and no drop in renal function related to both agents. Additionally, for patients with HBV-related cirrhosis, Koklu *et al.* [18] concluded that TDF and ETV were similarly safe agents for long-term use. Furthermore, Dogan *et al.* reported that both drugs were well tolerated with minimal side effects. No significant increase in creatinine was detected. [19]

### Virological breakthrough and resistance

Two studies referred to virological breakthrough or resistance. In the resistance surveillance by Liaw *et al.* [16], 13 patients (eight TDF, two emtricitabine [FTC]/TDF, and three ETV) qualified for genotypic testing based on viremia through 48 weeks, and no patient developed resistance to any study drug throughout the 48 weeks. Two of three ETV patients with baseline lamividine resistance switched to open-label FTC/TDF due to insufficient viral suppression at week 24 (all three had HBV DNA <400 copies/ml at week 48). Koklu *et al.* [18] reported that two of 77 patients switched from ETV to TDF at 24^th^ and 40^th^ month of treatment, respectively, because of virological breakthrough. No evidence for viral resistance to TDF was identified in these studies.

### Sensitivity analysis

Sensitivity analysis was performed via the random-effect model only for the 48 weeks HBV DNA suppression rate (the rest of indicators confined to limited articles are not concerned). Pooled RRs were similar before and after removal of each study, and no single study significantly altered the pooled RRs, suggesting the robust stability of these results (Table 4).

**Table 4:** Sensitivity analysis for the 48 weeks HBV DNA suppression rate.

Study omitted	RR	95% CI
Dogan, 2012	1.08	0.99–1.17
Gao, 2013	1.07	0.98–1.16
Koklu, 2013	1.10	0.99–1.22
Kurdas, 2011	1.07	0.98–1.17
Liaw, 2011	1.08	1.00–1.18
Sriprayoon, 2012	1.04	0.95–1.14
Combined	1.07	0.99–1.17

Note: RR, Relative Risk; CI, Confidence Interval.

### Publication bias

Funnel plot shapes revealed no evidence of asymmetry for all efficacy measures. Begg’s or Egger’s test also showed no publication bias (all *P* values > 0.05).

## Discussion

Entecavir (ETV) and tenofovir (TDF), two nucleotide analogs (NAs) are the most potent antiviral drugs for HBV infection [4]–[6]. Entacavir is a carboxylic analogue of guanosine that undergoes intracellular phosphorylation to its active 5’ triphosphate metabolite. This form competes with the natural substrate deoxyguanosine triphosphate to inhibit HBV DNA polymerase, which is essential for viral replication [26]. Likewise, Tenofovir undergoes phosphorylation to mimic deoxyguanosine 5’-triphosphate. Once incorporated into the HBV DNA polymerase reaction, it functions as a chain terminator. ETV and TDF share a similar mechanism to suppress HBV DNA – they both compete with native substrates for polymerase binding to terminate trascription [27]. Rates of HBV DNA suppression for TDF range from 68% to 90% and ETV from 61% to 92% after 48 weeks of therapy [28]–[32]. In our meta-analysis, results for pooled HBV DNA suppression rates for TDF and ETV were similar: 48 weeks post treatment were 80% and 76%, respectively, and 24 weeks post treatment were 50% and 46%, respectively. Furthermore, no significant differences in rates were seen between the TDF and ETV treatment groups (24 weeks: RR = 1.10 95% CI = 0.91–1.33; 48 weeks: RR = 1.07 95% CI = 0.99–1.17), suggesting similar efficacy for TDF and ETV in suppressing HBV DNA.

ALT level is a biomarker reflecting host immune response against virus-infected hepatocytes. ALT normalization usually follows a virological response and indicates cession of ongoing liver injury. In our meta-analysis, pooled ALT normalization rate for ETV was 76% and 75% for TDF. After 48 weeks of therapy, the pooled ALT normalization rate for ETV (81%) was similar to TDF (74%), indicating that both normalize ALT levels (24 weeks: RR = 0.89, 95% CI = 0.77–1.04; 48 weeks: RR = 0.91, 95%CI = 0.83–1.01).

Additionally, pooled HBeAg seroconversion for TDF was similar to the ETV group (24 weeks: 28% vs. 29%, RR = 0.86, 95% CI = 0.45–1.66; 48 weeks: 16% vs. 10%, RR = 1.09, 95% CI = 0.57–2.11). TDF and ETV do not greatly influence HBeAg seroconversion for HBV patients. Compared to pegylated interferon that enhances the host immune system to mount defense against HBV, oral NA therapy (including TDF and ETV) confers low HBeAg seroconversion rates at the end a year of treatment [33]. Furthermore, spontaneous seroconversion from HBeAg to anti-HBeAb during chronic hepatitis B is also immunologically mediated [3]. Therefore, it is plausible that TDF and ETV may have a marginal host immune system impact, yielding lower rates of seroconversion. This has yet to be supported.

Although oral nucleoside analogues, including ETV and TDF, are known to have relatively few side effects and are generally tolerated more than interferon, it is necessary to monitor long-term potential risks. ETV is classified as a category C drug and as it is associated with potential risk of fetal injury, it should be avoided during the first trimester of pregnancy [34]. TDF is eliminated mainly through nephrotoxicity [35], so all patients receiving TDF, creatinine clearance must be determined before and during therapy. Renal failure was not observed in Phase III clinical trials of TDF in patients with HBV monoinfection after up to 192 weeks of treatment [36]–[37]. Observation periods to date been too short, resulting in insufficient data to appraise whether difference exists for safety profiles of ETV and TDF.

To achieve long-term antiviral success, a high barrier to resistance is also critical for antiviral agents [38]. TDF and ETV both present low rates of resistance and have had success in patients failing to previous NA therapy [39]–[40]. Although resistance to ETV requires three mutations, pre-existing LAM resistance-associated mutations provide some foundation for ETV resistance [41]–[42] since resistance to ETV shares two common mutations (rtM204V and rtL180M) with LAM. In patients with LAM-resistant HBV, a high 6-year resistance rate of 57% has been suggested for ETV [10], [43]. Undetectable HBV DNA is not always achieved and virological breakthrough has occurred with ETV [44]. Additionally, it has been reported that sequential monotherapy of ETV can further promote multidrug resistant mutations [45]. Therefore, ETV monotherapy no longer be considered an optimal first-line therapy against LAM-resistant HBV. Moreover, TDF is a beneficial alternative for LAM failure patients, despite an incomplete resistance profile [13], [38], [39].

Some limitations merit consideration. In our study, major included studies were non-RCTs (5 of 7 studies). It has been reported that some factors, geographic, ethnic or disease status (CHB or cirrhosis) differences are possibly associated with agent efficacy. However, considering limited studies numbers for each factor, further analysis was restricted. Besides, due to the limited number of studies, analysis for some effect indicators might be underpowered (The power for 24 and 48 weeks HBV DNA suppression rates were 92.08% and 85.03%, respectively. The rest of indicators’ powers are less than 80%). Although current related studies have shown TDF may be used as an alternative agent against HBV infection in drug safety and resistance, this study results still need more studies and reasonable statistic methods used to explore safety and tolerability of these drugs.

Our meta-analysis indicates that ETV and TDF are comparable in efficacy and safety to sustain HBV DNA suppression with limited side effects. However, in considering limited efficacy of ETV in patients with LAM resistance, TDF is an alternative agent against HBV infection. Nonetheless, long-term efficacy and safety of TDF and ETV should be monitored in prolonged therapy in well-designed prospective studies with large sample sizes.

## Supporting Information

Checklist S1 **PRISMA Checklist.**
(DOC)
